# “Mind 4 Partner Abuse” Task: Assessment of Cognitive Patterns in Young Adults and Their Romantic Relationship Perceptions

**DOI:** 10.3390/bs16010004

**Published:** 2025-12-19

**Authors:** Silvia Mammarella, Laura Giusti, İmran Gökçen Yılmaz-Karaman, Anna Salza, Massimo Casacchia, Rita Roncone

**Affiliations:** 1 Department of Life, Health and Environmental Sciences, University of L’Aquila, 67100 L’Aquila, Italy; silvia.mammarella@univaq.it (S.M.); laura.giusti@univaq.it (L.G.); massimo.casacchia@univaq.it (M.C.); 2 Department of Psychiatry, Faculty of Medicine, Eskişehir Osmangazi University, Eskişehir 26040, Turkey; imrangokcen.yilmazkaraman@ogu.edu.tr; 3 University Unit for Rehabilitation Treatment, Early Interventions in Mental Health, S. Salvatore Hospital, 67100 L’Aquila, Italy; anna.salza@univaq.it

**Keywords:** instrument to assess toxic romantic relationships, gender-based violence, cognitive style, university students, university counseling services, psychoeducational interventions, gender-based violence vignette task

## Abstract

Toxic romantic relationships, a popular term referring to intimate partner violence (IPV) characterized by psychological, physical, and sexual violence, are a growing concern among young people. This pilot study aimed to preliminarily validate the vignette task on IPV, the “Mind 4 partner abuse” task, and to investigate the cognitive patterns and emotional profiles concerning IPV. Our research involved 228 university students from the University of L’Aquila who participated in an online psychoeducational program to raise awareness of the risks of IPV. Participants completed the “Mind 4 partner abuse” task, which included five vignettes depicting escalating violence in relationships. The task assessed participants’ emotional responses (anger, anxiety/fear, sadness, shame/guilt) and cognitive responses (functional-assertive or dysfunctional) to each vignette. In addition, for convergent validation, the Interpersonal Reactivity Index (IRI) was administered to assess empathic abilities. Five distinct factors were identified: active coping and legal awareness (ACLA), emotional distress (ED), assertiveness and autonomy defense (AAD), assertive reaction and self-empowerment (ARSE), and refusal of public humiliation and dignity assertion (RDA). One factor out of the five, emotional distress (ED), identified a dysfunctional cognitive pattern. The instrument showed a good convergent validity with the IRI. The correlation analysis showed that the IRI personal distress scale was negatively associated with ACLA and positively associated with ED. The IRI Empathic Concern scale was positively associated with RDA. In the dysfunctional cognitive pattern, as measured by the “Mind 4 Partner Abuse” vignette task, the ED factor was positively correlated with anxiety, sadness, shame, and guilt. The potential of the vignette task to identify high-risk cognitive profiles is promising, but it has yet to be confirmed. Given the limitations of the study, the findings offer only preliminary indications of cognitive patterns in young adults and their perceptions of romantic relationships, as assessed through a psychoeducational intervention. Further research with larger and more diverse samples, as well as more robust task designs, is necessary before firm conclusions can be drawn.

## 1. Introduction

The term “toxic”, when applied to romantic relationships, has become trendy on social media and in everyday language. It is a popular expression that refers to intimate partner violence (IPV), defined as any violence (physical, sexual, and/or emotional) that occurs within an intimate relationship (Robertson et al., 2020). Toxic romantic relationships have emerged as a significant social and public health problem that has captured the interest of many researchers, especially in recent decades.

A toxic romantic relationship consistently undermines one’s sense of well-being, happiness, and, at times, safety. Recent findings support the idea that violence in toxic intimate relationships may form part of a continuum whereby individuals experience and/or perpetrate such violence from adolescence through to adulthood (Piolanti et al., 2023). IPV in young relationships has serious negative mental and psychological consequences beyond immediate emotional and/or physical harm (Bonomi et al., 2013; Exner-Cortens et al., 2013; Park & Kim, 2018). IPV and sexual harassment are widespread in university settings (Kaufman et al., 2019), and demonstrate the severe physical, behavioral, academic, and/or mental health consequences of sexual victimization. Victimization appears to be long associated with depressive/anxiety symptoms and suicidal ideation/behavior (Piolanti et al., 2023; Puig-Amores et al., 2022; Torres Garcia et al., 2021). The prevalence of intimate partner violence (IPV) among university students varies considerably, ranging from 5% (Musa et al., 2021) to more than 50%. In fact, according to the UniSAFE report (Lipinsky et al., 2022), conducted in 46 universities in 15 European countries (N = 42,186), 58% of university students have experienced at least one form of gender-based violence—most often psychological (57%) or sexual harassment (31%)—highlighting the widespread diffusion of the phenomenon in European academic institutions. A study conducted with women students at an urban university in New York revealed that college attendance can both increase and decrease IPV risk: the risk rises when a partner attempts to control or restrict the student’s academic experience, but may decrease due to the opportunities and support provided by the university context (Tsui & Santamaria, 2015). The study of the relationship between IPV and academic difficulties showed that victims experience greater academic challenges and lower levels of campus belonging and perceived safety; these factors partially mediate the impact of IPV, with variations according to gender, ethnicity, and sexual orientation (Gezinski et al., 2025).

Most of the IPV literature has focused almost exclusively on adult couples. In recent years, research has shown that emerging adults are increasingly entering long-term intimate relationships at earlier ages, marked by episodes of conflict and prevarication (Claussen et al., 2022; Malhi et al., 2020; Menesini & Nocentini, 2008).

In Italy, in 2024, 16,117 women reported incidents of violence. Among them, 20.9% (3375 women) were between the ages of 18 and 30, and 8.3% (1336 women) were university students (Istituto Nazionale di Statistica, 2025). That same year, there was a 32.5% increase in calls for help related to stalking compared to the previous year, 2023. However, a broader analysis of data from both years reveals a significant rise in reports involving former partners: a 47.1% increase for former cohabitants, a 15.8% increase for ex-partners, and a 44.2% increase for lovers. This surge may partly account for the rise in requests for help with stalking.

Despite advances in equal opportunities and rights for men and women (López-Martínez et al., 2022; Tomlinson, 2011), ‘traditional’ or ‘rigid’ gender roles remain pervasive in Europe and worldwide, among both adults and young people. Traditional attitudes toward women and gender roles increase the likelihood of women being victims of partner aggression and men perpetrating partner violence (Garcia-Diaz et al., 2020). Particularly, girls exposed to societal norms based on sexist beliefs tend to idealize toxic romantic relationships disproportionately, have misconceptions about love, and normalize inequity and dysfunctional behaviors. The current scientific literature indicates that girls and boys hold attitudes associated with dominance in dating and relationships, driven by values and role expectations (Pastor-Bravo et al., 2023).

According to the cognitive–behavioral model (A. Beck, 1995), cognitions affect emotions that influence behavior, so cognitive and affective factors can predict men’s violent behavior and women’s limited readiness to terminate intimate partner violent relationships. A problematic thinking style related to catastrophizing, which involves exaggerating events or their consequences, was shown to be positively and strongly associated with violent behavior, especially for males (Bao et al., 2016). Toxic interpersonal relationships rely on maladaptive beliefs related to the conviction that “interpersonal relationships are unstable and insecure and are exposed to the risk of humiliation and harm” (Sojta et al., 2023). Additionally, in women, self-blame thoughts and feelings of shame and guilt are factors that lead to staying in toxic relationships with traumatic psychological consequences (J. G. Beck et al., 2011, 2015). In a student population, dysfunctional beliefs in romantic relationships were associated with mental and physical victimization, as well as poorer problem-solving skills (Kaygusuz, 2013).

Younger adults may be more prone to falling into dysfunctional thoughts related to toxic love due to factors such as inexperience in relationships, social pressure, and the search for peer approval. Recent data highlight a curvilinear pattern in which dysfunctional thoughts and attitudes in intimate relationships are pervasive during adolescence and young adulthood, then decrease with age due to factors such as greater self-confidence and the need for stable relationships based on trust and respect (Barnes et al., 2023). Psychoeducational interventions aimed at reducing the acceptance of couple violence and increasing awareness of emotional abuse showed positive effects sustained over time (An et al., 2024; Condino et al., 2016; Yalnizca-Yildirim & Cenkseven-Önder, 2025).

Empathy is positively associated with relationship quality (Ulloa & Hammett, 2016). The cognitive component of empathy (i.e., perspective-taking) significantly inhibits interpersonal aggression in an experimental setting (Richardson et al., 1994). In contrast, low empathy is associated with dating and sexual violence among adolescents and male college students (McCloskey & Lichter, 2003). In a sample of female college students, empathic abilities were identified as a significant predictor of dating victimization, and specifically “perspective taking” for psychological victimization and empathic concern for sexual victimization (Dodaj et al., 2020). Some Italian studies have attempted to assess university students’ empathetic attitudes toward IPV. Still, often, this is done through tools that lack interactivity, which tends to foster a sense of detachment from the phenomenon among young people (Rollè et al., 2018).

Based on the cognitive–behavioral model, within a psychoeducational intervention, this study aimed (1) to preliminarily validate the “Mind 4 partner abuse” vignette task on IPV and (2) to investigate the cognitive patterns and emotional profiles concerning IPV measured by the task. The instrument was administered in the context of a psychoeducational intervention as a means of demonstrating to young people that negative emotions and psychological distress are tied to dysfunctional, not assertive, thinking styles that can be changed.

Based on the theoretical framework and previous empirical findings, the following hypotheses were formulated.

- It was expected that the Mind 4 Partner Abuse task factor analysis would identify factors reflecting specific cognitive and emotional patterns associated with proneness to intimate partner violence (e.g., dysfunctional cognitions, empathy, guilt/shame, anger/hostility).
- For the convergent validity of the “Mind 4 Partner Abuse” vignette task, we employed the Interpersonal Reactivity Index (IRI) (Albiero et al., 2006; Davis, 1983). The factors identified through the Mind 4 Partner Abuse task are hypothesized to exhibit positive correlations with the IRI subscales assessing cognitive empathy (Perspective Taking) and affective empathy (Empathic Concern), and negative correlations with the Personal Distress subscale.

## 2. Materials and Methods

### 2.1. Participants

The present correlational pilot study was promoted and conducted by the University Counselling and Consultation Service for Students (SACS) at the University of L’Aquila, Italy. The SACS, founded in 1991, aims to support and help university students experiencing emotional difficulties due to academic failure or psychological distress.

The inclusion criterion was being a university student enrolled in medical and health professions courses within the Life, Health, and Environmental Sciences Department at the University of L’Aquila. The Department of Life, Health and Environmental Science of the University of L’Aquila manages 17 study programs (7 master’s degree courses and 10 degree courses) in 3 areas—medical, biological, and environmental sciences—with 2588 students enrolled in A.Y. 2020–2021 and with a teaching staff of 134 tenured teachers.

The decision to select students enrolled in medical and health courses (around 75% of the total student sample) within the Life, Health, and Sciences Department was based on their accessibility and relevance to the study’s focus on intimate partner violence (IPV) awareness and psychoeducation. These students are likely to encounter related issues in their future professional roles.

Participants were recruited via direct advertising on university websites and announcements of this study in an online psychoeducational short intervention lasting 4 h on gender and medicine, held on Women’s Day, 8 March 2021.

The main participants’ socio-demographic data were collected. The participants gave their informed consent, and information on this study and privacy protection was provided, ensuring the total confidentiality of the personal data, as required by the European General Data Protection Regulation n. 2016/679 no 181.

The students were invited to access the SACS service if they experienced severe emotional difficulties.

Participants did not receive any compensation for participating in this study. Ethical approval was obtained by the Internal Review Board of the University of L’Aquila (20 May 2020, n. 20/2020).

The sample included a total of 228 university students (around 12% of the sample belonging to the School of Medicine and Health Professionals degree courses of the University of L’Aquila), with a higher proportion of women (78.8%), who were statistically significantly younger than their male colleagues (men mean age: 25.8 SD 5.7; women mean age: 23.5 SD 5.6; ANOVA: F = 6.227; p = 0.013). The socio-demographic characteristics of the sample are reported in Table 1.

An a priori power analysis, based on the observed effect size for gender differences (Cohen’s d = 0.41), an α level of 0.05, and a desired statistical power of 0.80, indicated that approximately 100 participants per group (≈200 total) would be required. Our total sample of 228 participants meets this threshold, although the male subgroup remains smaller than optimal.

### 2.2. Affective Relationship Online Psychoeducational Intervention

All participants completed every module of the presented psychoeducational intervention. This structured and modular intervention was designed to increase awareness, promote functional skills, and support participants in recognizing and managing violence in intimate relationships. The topics of the brief psychoeducational intervention are reported in Table 2.

During the intervention, at the beginning of Module 2, the students were asked to complete the Socrative items regarding their primary socio-demographic data and to answer the experimental 5-vignette task guided by the researcher. In addition, the Interpersonal Reactivity Index (IRI) was administered to assess empathic ability. At the end of Module 2, the researchers presented the results of the Mind 4 Partner Abuse and discussed them with the participants, offering positive, constructive feedback.

### 2.3. Assessment Task: Mind 4 Partner Abuse

The assessment task “Mind 4 partner abuse” was developed by a focus group following the best practices for conducting and analyzing online focus groups described by Lobe (2017). We conducted three synchronous online focus group sessions on the platform. Each focus group session lasted approximately 60–120 min and included 10 participants, recruited voluntarily through the SACS counseling service.

The focus groups addressed the following aims: (1) in the first session, the students selected five IPV situations they deemed most significant; (2) in the second, they identified the key thoughts and emotions tied to the vignettes; and (3) in the third, they performed a final evaluation of the five “Mind 4 partner abuse” vignettes.

The five-vignette task “Mind 4 partner abuse” depicts different ways for a boy to disrespect his girlfriend or act violently towards her, according to a progressive intensity of violence: psychological and physical violence, stalking, and revenge porn (Supplementary Materials). The task was administered anonymously, which helps minimize social desirability bias toward the teacher-researcher.

**Figure 1:**
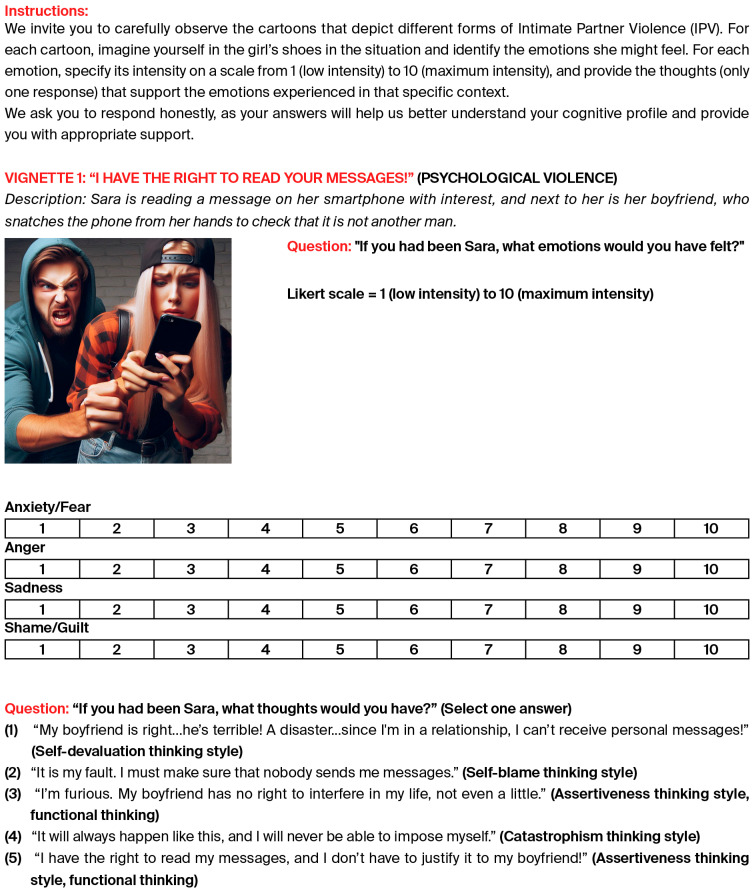
Presentation of Vignette 1 of the Mind 4 Partner Abuse task.

The task includes pictures showing interactions between young partners, inviting the subject (both women and men) to assume the victim’s perspective, and selecting the affective and cognitive responses that he/she would have felt/assumed in those situations (Figure 1 shows Vignette 1 of the Mind 4 Partner Abuse task).

Therefore, the task was to ask the subject to put himself in the girl’s shoes in the vignette to investigate “her” emotions (anger, anxiety/fear, sadness, shame/guilt) and thoughts. The subjects were invited to score on a 10-point Likert scale (1–10) the intensity of their feelings and related thoughts.

For each vignette, as defined in the focus group sessions, five sentences were identified and selected based on functional thinking (centered on self-respect, autonomy, and open requests for help for threats or violence) or dysfunctional thinking (statements attributed to anger, anxiety/fear, sadness, or shame/guilt).

The experimental task was presented one vignette at a time through Socrative^®^ (Showbie Inc., Edmonton, AB, Canada), a platform for online formative assessments used during the psychoeducational intervention.

Participants spent about 20 min completing the task.

### 2.4. Interpersonal Reactivity Index (IRI)

Our “Mind 4 Partner Abuse” vignette task was not previously used in validation studies. To preliminarily investigate the convergent validity of the instrument, we included the Interpersonal Reactivity Index (IRI) (Albiero et al., 2006; Davis, 1983) in our study. The IRI is a 28-item self-report instrument rated on a five-point Likert scale (from 1 = never true to 5 = always true) that investigates the cognitive and affective components of empathy. The cognitive dimension includes the “perspective-taking” subscale (PT), which assesses the tendency to adopt the psychological viewpoint of others spontaneously, and the “fantasy scale” subscale (FS), which evaluates preferences to transpose themselves imaginatively into the feelings and actions of fictitious characters in books, movies, and plays. On the other hand, the affective dimension includes the “empathetic concern” subscale (EC), which assesses “other-oriented” feelings and concerns for unfortunate others, and the “personal distress” subscale (PD), which measures “self-oriented” feelings of discomfort and negative activation in others’ difficult or emergency interpersonal situations. Each dimension includes seven items with possible scores ranging from 7 to 35. Cronbach’s alpha values for IRI subscales range from 0.70 to 0.83, and correlation coefficients from 0.01 to 0.37 between subscales (Davis, 1983; Albiero et al., 2006). Internal consistency for the IRI was high in the sample for the whole measure (Cronbach’s α = 0.80), and within the two affective dimension subscales (empathic concern α = 0.80; personal distress α = 0.78). Lower values were calculated for the two cognitive IRI subscales (perspective taking α = 0.64; fantasy scale α = 0.56).

This instrument was selected for the convergent validity of the “Mind 4 Partner abuse” vignette task because it was possible that empathy measured by the IRI would be positively associated with empathy toward the “victimized women” depicted in the vignettes. More relevant instruments for measuring the same construct were unavailable or could not be considered reference tools (i.e., the possible differences could not be attributed to the new instrument’s inferiority, especially given the original inclusion of images).

### 2.5. Statistical Analysis

A descriptive analysis of the sample’s socio-demographic characteristics was carried out.

Factor analysis was performed to validate the “Mind 4 Partner abuse” vignette task. Principal Component Analysis (PCA) with Varimax rotation was used to identify latent constructs. Initial testing included four-, five-, and six-factor solutions. The five-factor solution was ultimately selected based on eigenvalues (>1), scree plot inspection, explained variance, and the interpretability of the component structure. Items with loadings ≥ |0.30| were considered meaningful; cross-loadings and communalities were evaluated to ensure conceptual clarity. To assess the convergent validity of the “Mind 4 Partner Abuse” vignette task, a correlation analysis was conducted between the extracted factors and the four IRI subscale scores.

Compared to the 4- and 6-factor solutions, the selected 5-factor solution appeared to be the best, as it provided clearer identification of the extracted factors related to our cognitive model. The 5-factor model accounted for a sufficient proportion of total variance (43.5%) and showed conceptually coherent factors. The 4-factor extraction model accounted for 36.7% of the total variance, and the 6-factor extraction model accounted for 49.6%; however, the extracted factors were less clearly related to our model.

Gender differences in the means of the extracted factors were assessed using an ANOVA for continuous variables.

A correlation analysis between the extracted factors and emotional profiles was conducted to determine if cognitive patterns are positively associated with negative emotions. Statistical analyses were performed using SPSS 26.0 (SPSS Inc., Chicago, IL, USA).

## 3. Results

### 3.1. Emotion Intensity Reported in the “Mind 4 Partner Abuse” Vignette Task

**Figure 2:**
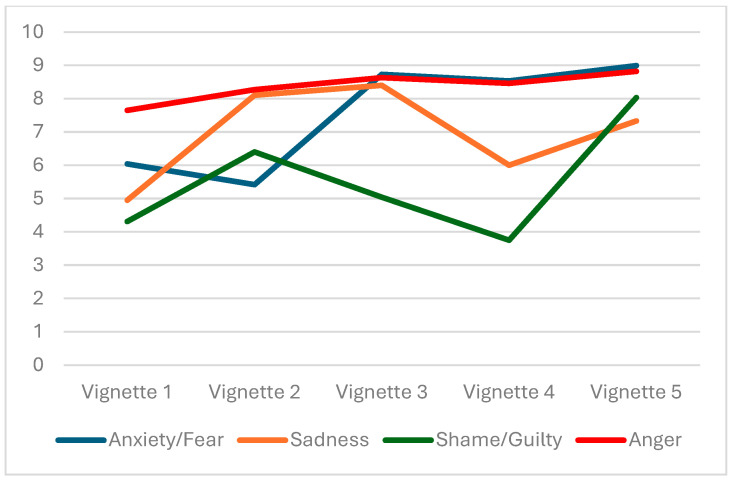
Mean scores of emotion intensity showed in the 5 Vignettes of the Mind 4 Partner Abuse task.

The intensity of the four emotions elicited by the Vignettes included in the “Mind 4 Partner abuse” vignette task is illustrated in Figure 2.

In Vignette 1, participants predominantly reported anger, followed by anxiety/fear. No statistically significant differences emerged between men and women for any of the emotions, suggesting a broadly similar emotional response across genders in this scenario. The emotional pattern shifted notably in Vignette 2. Anger remained the dominant emotion, but both sadness and shame/guilt increased, while anxiety/fear dropped to its lowest level across all vignettes. In Vignette 3, anxiety/fear and anger were almost equally prominent, with sadness also scoring relatively high. For Vignette 4, anxiety/fear emerged as the most salient emotion, followed closely by anger, while shame/guilt reached its lowest level among all vignettes. Finally, Vignette 5 elicited strong emotional responses across the board. Anxiety/fear was reported at the highest level, followed closely by anger.

Overall, these findings illustrate that while anger is a consistently dominant emotion across different relational scenarios, anxiety, sadness, and shame/guilt vary more depending on context. No statistically significant differences emerged between men and women for any of the emotions, suggesting a broadly similar emotional response across genders in these scenarios.

### 3.2. Preliminary Validation of the “Mind 4 Partner Abuse” Vignette Task

#### 3.2.1. Factor Analysis

Principal Component Analysis (PCA) with orthogonal Varimax rotation (Kaiser normalization) was performed on the 25 items of the “Mind 4 Partner Abuse” vignette task. This analysis aimed to reduce data dimensionality and identify the main components underlying adolescents’ experiences of relational control, emotional distress, and agency in romantic or peer digital contexts. The solution converged after six iterations.

The five distinct factors identified by the rotated component matrix are reported in Table 3, along with their correlation coefficients. We considered the items to make a significant contribution to the factors if their loading was 0.30 or greater. Five items had loadings of 0.30 or lower and were not included in the extracted components, although they were reported in the table. The retained items and the five items removed are listed in Supplementary Materials.

The first component (11.9% variance) included four items and was defined as “Active Coping and Legal Awareness, ACLA”.

This factor reflects a rational, externalized response to abuse, including awareness of legal rights, the intention to seek support from institutions, and assertive boundary setting. The factor cluster’s statements reflect a fight-back response (threatening to involve cyber-police, telling parents, and pursuing legal action). The mobilization of external resources, such as seeking support from family, peers, or the police, and the readiness to take action, indicates a form of problem-focused coping and legal consciousness that can be attributed to a “Functional cognitive style.”

The second component (9.5% variance) comprises six items and describes the “Emotional Distress, ED” dimension. Items loading strongly on this dimension express persistent fear (e.g., “I’m *scared… he threatens me*”, “*I feel tense and shaken*”) and a pervasive sense that the situation will never improve, suggesting a catastrophizing cognitive style. This component captures psychological distress, helplessness, fear of escalation in the face of threatening or coercive behavior, and proneness to expect ongoing abuse and self-blame. This component could reflect the cognitive aspects of “Devaluation”, “Self-blaming”, and “Catastrophizing” styles, as identified in our original hypothesis.

The third component (7.8% variance) is “Assertiveness and Autonomy Defense, AAD” and includes four items. Defined by anger-driven, autonomy-asserting statements, this factor embodies assertiveness, identity protection, and strong opposition to relational control, particularly jealousy and surveillance. Our original model defines this “active-anger” style as a “Functional cognitive” style.

Extremes in privacy control and dignity demands characterize the fourth component (variance 7.3%), “Assertive Reaction and Self-Empowerment, ARSE,” which includes three items and suggests a cognitive style that is not prone to accepting violent attitudes in intimate relationships. The items reflect assertive responses and rights-based language, such as “*I have the right…*” and “*he must not be allowed…*”. This factor likely represents self-assertion and personal agency in the face of relational threats. In our model, this factor could also be referred to as a “Functional cognitive style”.

The fifth component (6.8% variance) includes two items and identifies the dimension “Refusal of Public humiliation and Dignity Assertion, RDA”. This dimension indicates active resistance to public mistreatment and willingness to end the relationship if disrespect persists. It characterizes an assertive response and a strong sense of refusal to public humiliation. In our model, this factor could also be referred to as a “Functional cognitive style”.

No statistically significant gender differences were found for the five extracted factors.

#### 3.2.2. Inter-Factor Correlations (Pearson’s r)

Pearson’s r among the five factors indicated a strong negative relationship between active coping and legal awareness (F1), emotional distress (F2), and assertiveness and autonomy defense (F3), and a moderate positive relationship with the dimension of refusal of public humiliation and dignity assertion (F5). The emotional vulnerability dimension (F2) showed a strong negative relationship with assertiveness and autonomy defense (F3) and refusal of public humiliation and dignity assertion (F5), and a moderate negative relationship with assertive reaction and self-empowerment (F4) (Table 4).

#### 3.2.3. Convergent Validity of the “Mind 4 Partner Abuse” Vignette Task

To assess convergent validity of the “Mind 4 Partner abuse” vignette task, a correlation analysis was conducted between the extracted factors and the four IRI scale scores (Table 5).

The IRI personal distress scale showed a statistically significant negative relation with the active coping and legal awareness dimension and a positive statistically significant correlation with the emotional distress factor. The empathetic concern scale showed a statistically significant positive relation with the dimension of refusal of public humiliation and dignity assertion (Table 5).

#### 3.2.4. Correlation of the Extracted Five Factors and the Self-Assessed Emotions Referred to the “Mind 4 Partner Abuse” Vignette Task

Concerning the first vignette of the “*Mind 4 Partner abuse*” vignette task (*“I have the right to read your messages!”, psychological violence*) the correlation analysis between the four emotions (anger, anxiety/fear, sadness, shame/guilt) and the five extracted factors showed a negative relationship between anxiety/fear and assertiveness and autonomy defense, and refusal of public humiliation and dignity assertion (Table 6).

Concerning the second vignette (*“You’re worthless! What do you know, you’re just a woman”, psychological violence*), refusal of public humiliation and dignity assertion negatively correlated with anxiety/fear and sadness (Table 7).

The third vignette *(“Let’s not make drama…it’s just a slap!”, physical violence)* showed no statistically significant correlation between the five factors identifying the cognitive patterns and the four self-reported emotions inspired by the vignette (Table 8).

In the fourth vignette *(“Stalking with unwelcome and intrusive messages,” stalking)*, emotional distress was positively correlated with anxiety/fear. In contrast, active coping and legal awareness were negatively correlated with shame/guilt (Table 9).

In vignette 5 *(“You can’t leave me! Revenge with pictures”, revenge porn), active coping and legal awareness negatively correlated with anxiety/fear and shame/guilt*. Emotional distress was positively correlated with anxiety/fear, sadness, and shame/guilt (Table 10). Assertive reaction and self-empowerment were positively associated with anger. Refusal of public humiliation and dignity assertion was negatively correlated with sadness and shame/guilt.

## 4. Discussion

This pilot study aimed to validate the “Mind 4 partner abuse” task, which was about the cognitive patterns concerning IPV in a sample of university students enrolled in medical and health professions exposed to scenarios of IPV, and to identify the underlying thinking styles that may foster adaptive patterns or, conversely, heighten vulnerability to psychological harm in abusive relational contexts.

The psychometric validation of the “Mind 4 partner abuse” task was a complex and demanding job. The tool was implemented as an integrated instrument, assembling images and assessing emotion-intensity reactions and cognitive thinking styles.

Concerning the questions tied to the thinking styles, the exploratory factor analysis revealed a solid five-factor structure which aligns with our theoretical framework—active coping and legal awareness (ACLA), emotional distress (ED), assertiveness and autonomy defense (AAD), assertive reaction and self-empowerment (ARSE), and refusal of public humiliation and dignity assertion (RDA). These dimensions differentiate between functional cognitive styles (e.g., problem-focused coping, assertiveness, legal consciousness) and dysfunctional styles (e.g., emotional distress, catastrophizing, self-blame), whereas the definitions of “functional” and “dysfunctional” refer to the cognitive-behavioral model and the associated thinking styles. The proportion of explained variance of our model (43.5%) cannot be considered entirely satisfactory. However, the five-factor solution offers a nuanced understanding of how university students experience and cognitively process emotional and digital IPV scenarios. The factors range from fear-based responses to proactive legal strategies, self-devaluation, and public assertiveness, capturing the multidimensional nature of victim responses.

Correlations with the IRI scale further support the tool’s convergent validity: the IRI personal distress scale was positively correlated with the ED factor and negatively correlated with ACLA. In contrast, the IRI empathic concern scale was positively associated with RDA, suggesting that moral rejection of public humiliation may be rooted in empathic sensitivity.

Concerning the emotions elicited by cognitive styles, the most identified factors in our tool, referred to as “positive thinking styles”, centered on active coping, assertiveness, refusal of public humiliation, and self-empowerment. They were negatively associated with anxiety and fear, sadness, and shame and guilt, and positively associated with anger. In this case, we could hypothesize that such emotions will not drive violent behaviors (Caldwell et al., 2009), but help young people not to be passive recipients of IPV. The dysfunctional thinking style identified as the “Emotional Distress” factor in the Mind 4 Partner Abuse was positively correlated with anxiety and fear concerning the more intense violent vignettes showing stalking and revenge porn. In the latter, sadness, shame, and guilt seem to be emotional consequences of helplessness, fear of escalation in the face of threatening or coercive behavior, and proneness to expecting ongoing abuse and self-blaming.

The results of the present study showed that anger represents a dominant and relatively stable emotion across different relational contexts. In contrast, emotions, such as anxiety, sadness, and shame/guilt, appear to be more sensitive to situational variation. This evidence suggests that anger may constitute a more generalizable affective response within interpersonal dynamics, maintaining a transversal relational function—perhaps connected to evolutionary mechanisms of defense and assertion, and likely linked to perceptions of injustice or frustration that cut across different types of relationships (Srivastava & Bharti, 2018). The absence of statistically significant differences between men and women across the emotions considered is consistent with a growing body of research challenging the traditional view of “gendered emotional differentiation.” In fact, men and women experience the same amount of anger; the difference lies in how they express it (Srivastava & Bharti, 2018). Participants often underestimated psychological violence, whereas physical violence is perceived as more severe and elicits similar cognitive and emotional responses. This is consistent with recent literature showing that psychological IPV is often underestimated or unrecognized (Brennan et al., 2021; Cinquegrana et al., 2023).

Our results are promising and in line with the importance of screening young adults for issues related to gender-based violence (An et al., 2024). Specific IPV psychoeducational interventions offering such assessment could be the key to preserving the mental health of young people, as the scientific literature highlights a strong association between IPV and symptoms such as anxiety, depression, post-traumatic stress disorder, substance abuse, and chronic pain (An et al., 2024; Condino et al., 2016).

So far, very few scientific studies have explored cognitive styles in response to IPV vignettes among university students, and our study could stimulate future work to identify at-risk cognitive profiles in young individuals, suggesting that dysfunctional thinking styles may be associated with significant emotional distress. The use of a vignette task, such as the Mind 4 Partner Abuse, can represent a first and preliminary step to improve a methodological and ecological tool to identify early on the dysfunctional cognitive profiles of young people at risk of normalizing violence within toxic romantic relationships. In this study, we took an initial step by involving students enrolled in medical and health professions programs, where women represent a higher proportion of the student body—reflecting the ongoing “feminization of the medical profession” and of health-related fields in Italy (Vezzani, 2024). Although the results cannot be generalized to the broader emerging adult population, we believe they can be reliably extended to young students pursuing degrees in medical and health professions.

## 5. Strengths and Limitations

To the best of our knowledge, this is the first Italian study to develop a new vignette-task instrument to evaluate toxic intimate relationships—a practical, non-intrusive, and inexpensive tool to examine emotions, individual beliefs, and cognitions regarding the sensitive issue of aggressive behaviors in young couples. Using a vignette task could effectively allow for the study of such matters, especially when participants may feel uncomfortable discussing personal challenges or their answers may not be socially acceptable (Hess et al., 2016).

Despite the contributions of the present study, several limitations should be acknowledged.

First, the instrument’s convergent validity, assessed using the IRI, shows acceptable reliability in our sample, indicating that the measures tend to align with similar, already-validated constructs. However, the picture of divergent validity has not yet been explored: we did not test the instrument’s relationship with a general adverse effect or social desirability. This limitation in fully understanding the instrument’s validity highlights the need for future studies that include divergence measures to ensure the instrument assesses the constructs of interest without being influenced by extraneous factors.

Second, the psychometric evaluation could be further strengthened through split-sample analyses using EFA/CFA or ESEM approaches to validate the factor structure more robustly.

Third, criterion and predictive validity were not assessed in this study, and future work should examine the instrument’s ability to predict relevant behavioral or clinical outcomes over time.

Fourth, a more detailed investigation of measurement invariance across gender and age groups is needed to ensure that the instrument functions equivalently across different subpopulations. The overrepresentation of women could have compromised the test’s validity. Indeed, our data reflect the current gender distribution in Italian medical schools and health profession degree programs, where women represent 53.3% and 70.3% of students, respectively (ALMA LAUREA, 2019). Indeed, sample characteristics or contextual factors may account for the absence of gender differences, and future research should consider larger, more diverse samples to verify these findings.

Fifth, we adopted a gender-binary, monogamous approach to toxic romantic relationships. The authors did not include LGBTQIA+ populations to avoid privacy and coming-out issues in a relatively small university setting, despite the anonymity of this study. We should have examined toxic beliefs and behaviors in lesbian, gay, bisexual, transgender, and questioning (LGBTQIA+) populations as well. It could be that LGBTQIA+ relationships do not operate according to a heterosexual script (Kim et al., 2007; Whitfield et al., 2021).

## 6. Conclusions

The potential of the vignette task to identify high-risk cognitive profiles is promising, but it has yet to be confirmed. Given the limitations of the current study, the findings should be interpreted with caution. They offer only tentative insight into cognitive patterns in young adults, and future research will need to rely on broader sampling strategies and more rigorous task materials to obtain more reliable and generalizable evidence.

We hope that our study will stimulate future work and psychoeducational events aimed at using vignettes as a methodological and ecological tool to identify early on the dysfunctional affective and cognitive profiles of young people at risk of normalizing violence within a toxic romantic relationship, and raise awareness of all psychological and physical violent behaviors, even in non-heterosexual couples.

Finally, this study aligns with the United Nations’ Sustainable Development Goals, namely Goal 5. According to the United Nations (2015), this goal aims to achieve equal opportunities for women and men in economic development, the elimination of all forms of violence against women and girls (including the abolition of forced and early marriages), and equal rights at all levels of participation.

## Figures and Tables

**Table 1:** Socio-demographic characteristics of the sample.

Variables	University Students (n = 228)
Gender n (%)	
Male	49 (21.5)
Female	179 (78.5)
Age, mean (±SD)	24 (±5.6)
Accademic course (%)	
Second-Level Degree (unique 6-year cycle) Course in Medicine and Surgery	51 (22.4)
Second-Level Degree in Nursing and Obstetric Sciences	14 (6.1)
First-Level Degree Course in Nursing	61 (26.8)
First-Level Degree Course in Physiotherapy	22 (9.6)
First-Level Degree Course in Neuro and Psychomotor Childhood Therapy	19 (8.3)
First-Level Degree Course in Psychiatric Rehabilitation Technique	46 (20.2)
First-Level Degree Course in Speech Therapy	4 (1.8)
First-Level Degree Course in Orthoptics and Ophthalmological Assistance	10 (4.4)
First-Level Degree Course in Dietetics	1 (0.4)
Father education (%)	
Primary school diploma	5 (2.2)
Middle school diploma	45 (19.7)
High school graduation	135 (59.2)
University degree	43 (18.9)
Mother education (%)	
Primary school diploma	5 (2.2)
Middle school diploma	47 (20.6)
High school graduation	132 (57.9)
University degree	44 (19.3)

**Table 2:** Brief psychoeducational intervention model inspired by the DEEP ACTS project (Developing Emotional Education Pathways and Art-Centered Therapy Services against gender violence) project funded by the European program “Rights, Equality and Citizenship” spread over the two years, 2020–2022, in Italy, Portugal, and Spain.

	Module Content	Duration
Module 1	The module was centered on emotional education based on the cognitive-behavioral model and introduced attachment theory and its implications in intimate relationships.	20 min
Module 2	The module included an online compilation on the Socrative platform titled “Mind 4 partner abuse”, a vignette task designed to raise awareness and help recognize situations of partner abuse. At the end of the compilation, the results were presented and discussed with participants, providing positive, constructive feedback. Some results were revisited, as examples, in the following sections of the intervention.	20 min
Module 3	The module focused on defining functional intimate relationships, explaining intimate partner violence (IPV), and identifying IPV warning signs. This module also addressed different types of violence (psychological, physical, and social) and presents data on Italian samples.	20 min
Module 4	This module aims to help participants recognize dysfunctional thinking styles and learn how to think more functionally.	20 min
Module 5	Module 5 illustrated how to exit situations of violence and offers helpful information for support.	10 min

**Table 3:** Rotated component matrix (Varimax)—five-factor solution of the “Mind 4 Partner abuse” vignette task.

Vignette/ Item	Items Content	C1	C2	C3	C4	C5
4.2	Luca hasn’t accepted…report to cyber-police	**0.858**				
4.5	Relationship ended…not his property	**0.617**	−0.492			
5.1	No right to share photos…blackmail	**0.579**	−0.441			
5.4	Embarrassing…my fault for trusting him	**−0.506**				
3.1	Will always be like this…no respect		**0.667**			
4.4	Scared…he threatens me		**0.562**			−0.325
5.2	Trapped…life ruined forever		**0.440**			
2.1	Terrified every time I speak		**0.417**			
1.2	It’s my fault…must block messages		**0.324**			
5.3	Worth nothing…never thought he’d act like this		**0.306**			
1.1	A disaster…being engaged		0.285			
2.2	It’s always the same…never changes		0.279			
3.3	Right to go out with friends		−0.319	**0.842**		
3.5	Angry… can’t stand interference			**0.695**		
5.5	He’s a bastard… he’ll pay			**0.506**		
3.2	He might leave me…			**−0.418**		
1.4	Always like this… can’t stand up		0.279			
4.1	Rumors…easy girl		0.242			
1.5	Right to read my messages				**0.943**	
1.3	Angry…no interference				**0.896**	
4.3	Desperate…can’t enjoy life				**−0.373**	
2.5	Mustn’t treat me badly in public					**0.893**
2.4	Tired of mistreatment…end relationship					**0.882**
2.3	He maltreats me…but my fault					−0.159

Bold indicates statistical significance of the data.

**Table 4:** Inter-factor correlations (Pearson’s r) of the five factors extracted from the “Mind 4 Partner abuse” vignette task.

Factors	F1. ACLA	F2. ED	F3. AAD	F4. ARSE	F5. RDA
F1. Active Coping and Legal Awareness, ACLA	—	**−0.754** **	**−0.271** **	0.054	**0.149** *
F2. Emotional Distress, ED		—	**−0.332** **	**−0.140** *	**−0.347** **
F3. Assertiveness and Autonomy Defense, AAD			—	0.007	0.064
F4. Assertive Reaction and Self-Empowerment, ARSE				—	0.017
F5. Refusal of Public Humiliation and Dignity Assertion, RDA					—

* = *p* < 0.05; ** = *p* < 0.01. Bold indicates statistical significance of the data.

**Table 5:** Correlations between the extracted five factors and the four IRI scales.

IRI Subscale	F1. ACLA	F2. ED	F3. AAD	F4. ARSE	F5. RDA
IRI PT (Perspective Taking)	**0.105** *	−0.036	−0.074	0.058	0.086
IRI FS (Fantasy)	**0.111** *	−0.039	−0.056	0.019	0.045
IRI EC (Empathic Concern)	0.040	−0.039	0.006	**0.130** *	**0.179** **
IRI PD (Personal Distress)	**−0.282** **	**0.213** **	0.078	−0.046	−0.062

* = *p* < 0.05; ** = *p* < 0.01. F1. **ACLA**, Active Coping and Legal Awareness; F2. **ED**, Emotional Distress; F3. **AAD**, Assertiveness and Autonomy Defense; F4. **ARSE**, Assertive Reaction and Self-Empowerment; F5. **RDA**, Refusal of Public Humiliation and Dignity Assertion. Bold indicates statistical significance of the data.

**Table 6:** Correlations between the extracted five factors and the four emotions elicited in Vignette 1.

Factors	Anxiety/Fear	Sadness	Shame/Guilt	Anger
F1. Active Coping and Legal Awareness	0.040	0.012	−0.085	0.025
F2. Emotional Distress	0.073	0.031	0.089	−0.043
F3. Assertiveness and Autonomy Defense	**−0.141** *	−0.085	−0.029	0.066
F4. Assertive Reaction and Self-Empowerment	0.053	−0.003	0.039	0.057
F5. Refusal of Public Humiliation and Dignity Assertion	**−0.140** *	−0.004	0.056	−0.064

* = *p* < 0.05. Bold indicates statistical significance of the data.

**Table 7:** Correlations between the extracted five factors and the four emotions elicited in Vignette 2.

Factors	Anxiety/Fear	Sadness	Shame/Guilt	Anger
F1. Active Coping and Legal Awareness	−0.064	0.016	−0.074	−0.022
F2. Emotional Distress	0.092	0.015	0.108	0.043
F3. Assertiveness and Autonomy Defense	−0.035	−0.002	−0.022	−0.029
F4. Assertive Reaction and Self-Empowerment	0.074	−0.043	0.015	0.043
F5. Refusal of Public Humiliation and Dignity Assertion	−0.182 **	−0.142 *	−0.109	0.061

* = *p* < 0.05; ** = *p* < 0.01. Bold indicates statistical significance of the data.

**Table 8:** Correlations between the extracted five factors and the four emotions elicited in Vignette 3.

Factors	Anxiety/Fear	Sadness	Shame/Guilt	Anger
F1. Active Coping and Legal Awareness	−0.033	−0.056	−0.106	0.028
F2. Emotional Distress	0.088	0.045	0.126	−0.049
F3. Assertiveness and Autonomy Defense	−0.045	0.023	−0.083	0.053
F4. Assertive Reaction and Self-Empowerment	0.039	0.070	0.070	0.121
F5. Refusal of Public Humiliation and Dignity Assertion	−0.127	−0.112	−0.046	0.028

* = *p* < 0.05; ** = *p* < 0.01.

**Table 9:** Correlations between the extracted five factors and the four emotions elicited in Vignette 4.

Factors	Anxiety/Fear	Sadness	Shame/Guilty	Anger
F1. Active Coping and Legal Awareness	−0.089	−0.033	**−0.181** **	0.072
F2. Emotional Distress	**0.142** *	0.073	0.103	−0.047
F3. Assertiveness and Autonomy Defense	−0.077	−0.055	0.030	0.025
F4. Assertive Reaction and Self-Empowerment	0.012	0.024	0.012	−0.004
F5. Refusal of Public Humiliation and Dignity	−0.035	−0.081	0.095	−0.037

* = *p* < 0.05; ** = *p* < 0.01. Bold indicates statistical significance of the data.

**Table 10:** Correlations between the extracted five factors and the four emotions elicited in Vignette 5.

Factors	Anxiety/Fear	Sadness	Shame/Guilty	Anger
F1. Active Coping and Legal Awareness	**−0.161** *	−0.094	**−0.228** **	0.065
F2. Emotional Distress	**0.138** *	**0.134** *	**0.199** **	−0.099
F3. Assertiveness and Autonomy Defense	−0.014	−0.042	0.016	0.082
F4. Assertive Reaction and Self-Empowerment	**0.107**	0.081	0.024	**0.212** **
F5. Refusal of Public Humiliation and Dignity	−0.076	**−0.175** **	**−0.141** *	−0.072

* = *p* < 0.05; ** = *p* < 0.01. Bold indicates statistical significance of the data.

## Data Availability

The data supporting the conclusions of this article will be made available by the authors upon reasonable request.

## Supplementary Materials

The following supporting information can be downloaded at: https://www.mdpi.com/article/10.3390/bs16010004/s1, “Mind 4 partner abuse vignette task”.
