# Supplementary material for: “Mind 4 Partner Abuse” Task: Assessment of Cognitive Patterns in Young Adults and Their Romantic Relationship Perceptions
Source: Behav Sci (Basel). 2025 Dec 19;16(1):4. doi: 10.3390/bs16010004 (PMC12837563; doi:10.3390/bs16010004)
Supplement: Supplementary file 1 [file behavsci-16-00004-s001.zip › behavsci-3580305-supplementary.pdf]

“MIND 4 PARTNER ABUSE” TASK VIGNETTE

Instructions:
We invite you to carefully observe the cartoons that depict different forms of Intimate Partner Violence (IPV). For each cartoon, imagine yourself in the girl's shoes in the situation and identify the emotions she might feel. For each emotion, specify its intensity on a scale from 1 (low intensity) to 10 (maximum intensity), and provide the thoughts (only one response) that support the emotions experienced in that specific context.
We ask you to respond honestly, as your answers will help us better understand your cognitive profile and provide you with appropriate support.

VIGNETTE 1: "I HAVE THE RIGHT TO READ YOUR MESSAGES!" (PSYCHOLOGICAL VIOLENCE)
Description: Sara is reading a message on her smartphone with interest, and next to her is her boyfriend, who snatches the phone from her hands to check that it is not another man.

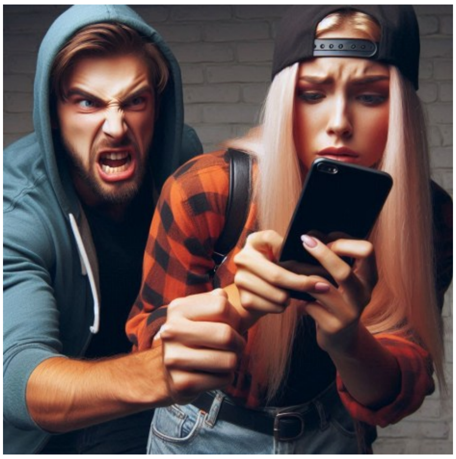

Question: "If you had been Sara, what emotions would you have felt?"

Likert scale= 1 (low intensity) to 10 (maximum intensity)

Anxiety/Fear

1	2	3	4	5	6	7	8	9	10

Anger

1	2	3	4	5	6	7	8	9	10

Sadness

1	2	3	4	5	6	7	8	9	10

Shame/Guilt

1	2	3	4	5	6	7	8	9	10

Question: " If you had been Sara, what thoughts would you have?" (Select one answer)

1) "My boyfriend is right… he's terrible! A disaster… since I'm in a relationship, I can't receive personal messages!" (**Self-devaluation thinking style)**

2) "It is my fault. I must make sure that nobody sends me messages" (**Self-blame thinking style)**

3) "I'm furious. My boyfriend has no right to interfere in my life, not even a little." (**Assertiveness thinking style, functional thinking)**

4) "It will always happen like this, and I will never be able to impose myself" (**Catastrophism thinking style)**

5) "I have the right to read my messages, and I don't have to justify it to my boyfriend!" (**Assertiveness thinking style, functional thinking)**

VIGNETTE 2: "YOU'RE WORTHLESS! WHAT DO YOU KNOW, YOU'RE JUST A WOMAN" (PSYCHOLOGICAL VIOLENCE)

Description: "Mauro always maltreats Veronica when they are with friends. He often despises and ridicules her because she contradicts him. His favorite sentence is "Shut up! You are worthless! What do you want to understand? You are a woman!"

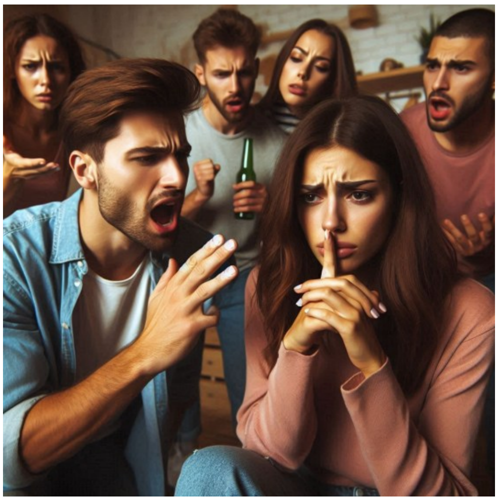

Question: "If you had been Veronica, what emotions would you have felt?"

Likert scale= 1 (low intensity) to 10 (maximum intensity)

Anxiety/Fear

1	2	3	4	5	6	7	8	9	10

Anger

1	2	3	4	5	6	7	8	9	10

Sadness

1	2	3	4	5	6	7	8	9	10

Shame/Guilt

1	2	3	4	5	6	7	8	9	10

Question: "If you had been Veronica, what thoughts would you have?" (Select one answer)

1) "Every time I open my mouth, I'm scared! I can't take it anymore. I feel agitated and shaken" **(Self-devaluation thinking style)**
2) "It's always the same thing… it will always be like this… the situation will never change..!" **(Catastrophism thinking style)**
3) "What a shame. He maltreats me in front of everyone. But it's my fault, I shouldn't contradict him" **(Self-blame thinking style)**
4) "I'm tired of being maltreated in front of our friends. I'm not afraid to be proven wrong! If you don't assure me that you will change this behavior, it is better to end this relationship immediately" **(Assertiveness thinking style, functional style)**
5) "He absolutely cannot maltreat me in front of everyone and make me look like an idiot!" **(Assertiveness thinking style, functional style)**

VIGNETTE 3: "LET'S NOT MAKE DRAMA...IT'S JUST A SLAP!" (PHYSICAL VIOLENCE)

Description: "Sergio feels very angry because he doesn't want his girlfriend Silvia to go out alone with her friends. So, after a discussion, he slaps her in the face... and it's not the first time it's happened!".

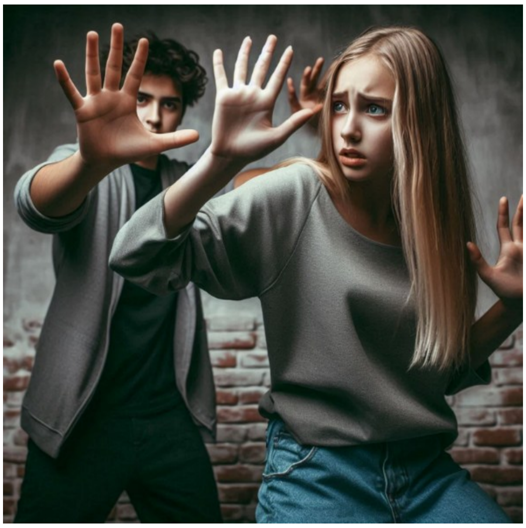

Question: " If you had been Silvia, what emotions would you have felt?"

Likert scale= 1 (low intensity) to 10 (maximum intensity)

Anxiety/Fear

1	2	3	4	5	6	7	8	9	10

Anger

1	2	3	4	5	6	7	8	9	10

Sadness

1	2	3	4	5	6	7	8	9	10

Shame/Guilt

1	2	3	4	5	6	7	8	9	10

Question: " If you had been Silvia, what thoughts would you have?" (Select one answer)

1) "It will always happen like this! He will never be okay with me going out with them! I will never be able to to assert myself" **(Catastrophism thinking style)**
2) "My boyfriend is right, he's terrible! Being in a relationship, I can't go out with my friends. He might leave me!" **(Self-devaluation thinking style)**
3) "I have the right to go out with my friends, and I don't have to ask my boyfriend's permission" **(Assertiveness thinking style, functional style)**
4) "It's my fault if he slapped me. I was wrong because I know he doesn't want me to go out with my friends. He warned me!" **(Self-blame thinking style)**
5) "I'm furious. I can't stand my boyfriend interfering in my life" **(Assertiveness thinking styles, functional style)**

VIGNETTE 4: "STALKING WITH UNWELCOME AND INTRUSIVE MESSAGES" (STALKING)

"Jessica and Luca ended a relationship that lasted 1 year two months ago. He is a very jealous and possessive and does not accept that Jessica is now interested in another guy. Every day Luca sends her many unpleasant and offensive messages on WhatsApp... even threats against her".

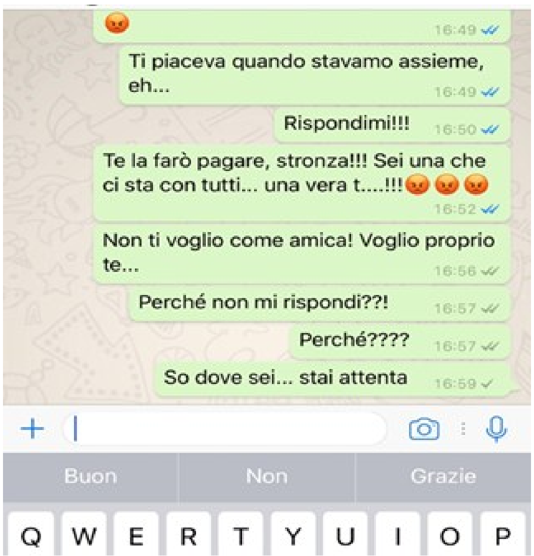

Question: "If you had been Jessica, what emotions would you feel?"

Likert scale= 1 (low intensity) to 10 (maximum intensity)

Anxiety/Fear

**1**	**2**	**3**	**4**	**5**	**6**	**7**	**8**	**9**	**10**

Anger

**1**	**2**	**3**	**4**	**5**	**6**	**7**	**8**	**9**	**10**

Sadness

**1**	**2**	**3**	**4**	**5**	**6**	**7**	**8**	**9**	**10**

Shame/Guilt

**1**	**2**	**3**	**4**	**5**	**6**	**7**	**8**	**9**	**10**

Question: " If you had been Jessica, what thoughts would you have?" (Select one answer)

1) "Woe is me! Luca will go around saying that I'm an easy girl...!" **(Self-blame thinking styles)**
2) "Luca hasn't accepted the end of our story, and he's very immature! I've been very understanding towards him, and I haven't blocked him, but now he's exaggerating! I'll report him to the postal police" **(Assertiveness thinking style, functional style)**
3) "I'm desperate! I don't dare to do anything... I will never enjoy life and my relationships with this torment... **(Self-devaluation thinking styles)**
4) "I'm scared...he writes terrible things; he threatens me all the time...what if he does what he says?" **(Catastrophism thinking style)**
5) "What has he gotten into his head???... our relationship ended a while ago. I am not his property! It would be better for him if he stopped" **(Assertiveness thinking styles, functional style)**

VIGNETTE 5:

"Mirella sent pictures of herself in her underwear to a boy she likes and with whom she no longer goes out. Now she is being blackmailed by the boy, who has not accepted being dumped, and who wants to share her intimate pictures on social networks."

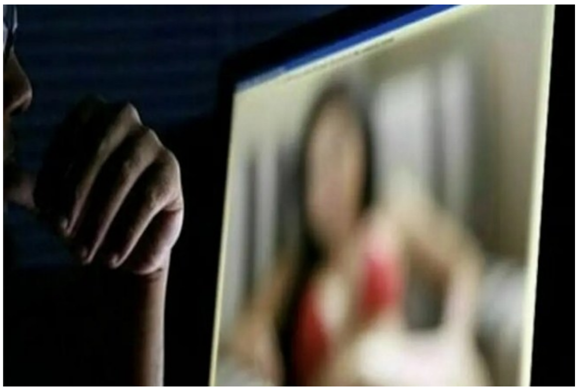

Question: "If you had been Mirella, what emotions would you feel?"

Likert scale= 1 (low intensity) to 10 (maximum intensity)

Anxiety/Fear

1	2	3	4	5	6	7	8	9	10

Anger

1	2	3	4	5	6	7	8	9	10

Sadness

1	2	3	4	5	6	7	8	9	10

Shame/Guilt

1	2	3	4	5	6	7	8	9	10

Question: " If you had been Mirella, what thoughts would you have?" (Select one answer)

1) "He has no right to spread my photos! I will tell my parents and friends. I will go to the police to report him for his blackmail. It will be very unpleasant, but it will not be the end of the world to tell my parents to help me" **(Assertiveness thinking style, functional style)**
2) "I was framed… I'm afraid of what would happen if the photos were to circulate… my life would be destroyed forever" **(Catastrophism thinking style)**
3) I didn't think he would behave like this... I've always been honest with him... I'm finished, I don't understand anything, I'm worthless!" **(Self-devaluation thinking style)**
4) "What a shame... I shouldn't have sent those photos… if my parents found out… it's my fault for trusting him" **(Self-blame thinking style)**
5) "I can't believe it! He's a bastard! He absolutely cannot behave like this! He needs to pay the price!" **(Assertiveness thinking styles, functional style)**
